# Polish validation of brace questionnaire

**DOI:** 10.1186/1748-7161-7-S1-O2

**Published:** 2012-01-27

**Authors:** E Kinel, T Kotwicki, A Podolska, W Stryła

**Affiliations:** 1 Department of Rehabilitation University of Medical Sciences and Rehasport Clinic Poznan, Poland; 2 Spine Disorders Unit, Department of Pediatric Orthopedics and Traumatology, University of Medical Sciences, Poznan, Poland; 3 Department of Rehabilitation, University of Medical Sciences, Poznan, Poland

## Background

The aim of the study was to undertake the process of cultural adaptation of the Brace Questionnaire (BrQ) into Polish. The BrQ is an instrument for measuring quality of life of scoliotic adolescents who are being treated conservatively with wearing of a corrective brace [1]. The BrQ consists of 34 Likert-scale items associated with eight domains.

## Material and methods

The translation from the original Greek into Polish was performed. The process of cultural adaptation of the questionnaire was compliant with the guidelines of the International Quality of Life Assessment (IQOLA) Project. It involved 30 adolescents, ages ranging between 10.0 and 17.0 years, all with Adolescent Idiopathic Scoliosis (AIS) and all wearing the same kind of brace (Chêneau). The statistical analysis calculated the reliability (internal consistency), floor and ceiling effects of the BrQ [2-5].

## Results

The age was 14.0 +/- 1.6 years. The adolescents were wearing the brace for more than 3 months. Cobb angle was 33.6 +/- 11.6 degrees. The internal consistency was satisfactory: Cronbach’s alpha coefficient was 0.82, p<0.001. There was no floor or celing effect.

## Conclusion

Polish version of BrQ is reliable and can be used in Polish adolescents with idiopathic scoliosis wearing the brace to assess their quality of life.
